# Mechanisms of Action of Antipsychotic Drugs of Different Classes, Refractoriness to Therapeutic Effects of Classical Neuroleptics, and Individual Variation in Sensitivity to their Actions: PART I

**DOI:** 10.2174/157015909790031229

**Published:** 2009-12

**Authors:** R Miller

**Affiliations:** Otago Centre for Theoretical Studies in Psychiatry and Neuroscience (OCTSPAN), Department of Anatomy and Structural Biology, School of Medical Sciences, University of Otago, P.O.Box 913, Dunedin, New Zealand

**Keywords:** Antipsychotic drugs, neuroleptic drugs, cholinergic interneurones, D1 receptors, D2 receptors, muscarinic M1 receptors, muscarinic M4 receptors, neuroleptic threshold, atypical antipsychotic agents.

## Abstract

Many issues remain unresolved about antipsychotic drugs. Their therapeutic potency scales with affinity for dopamine D2 receptors, but there are indications that they act indirectly, with dopamine D1 receptors (and others) as possible ultimate targets. Classical neuroleptic drugs disinhibit striatal cholinergic interneurones and increase acetyl choline release. Their effects may then depend on stimulation of muscarinic receptors on principle striatal neurones (M4 receptors, with reduction of cAMP formation, for therapeutic effects; M1 receptors for motor side effects). Many psychotic patients do not benefit from neuroleptic drugs, or develop resistance to them during prolonged treatment, but respond well to clozapine. For patients who *do *respond, there is a wide (>ten-fold) range in optimal doses. Refractoriness or low sensitivity to antipsychotic effects (and other pathologies) could then arise from low density of cholinergic interneurones. Clozapine probably owes its special actions to direct stimulation of M4 receptors, a mechanism available when indirect action is lost.

## 1. INTRODUCTION

The first antipsychotic drug - chlorpromazine - was discovered serendipitously in the early 1950s. It was soon realized that, as well as antipsychotic actions, such drugs produce motor side effects reminiscent of Parkinson’s disease. This combination of main effect and potent motor side effects led to their being called “neuroleptic drugs”. In the 1960s, it started to be understood that these medications act by blocking receptors for the neurotransmitter dopamine. By the early 1980s it was known that there is more than one type of dopamine receptor, and today these are divided into two classes - the D1-class receptor (D1 and D5), and the D2-class (D2, and its variants, plus D3 and D4). For much of this historical development, common understanding has been based on a number of implicit assumptions: (i) A single receptor type - the D2 receptor - is responsible for both the antipsychotic main effect, and the motor side effects; (ii) Antipsychotic action is relatively rapid, once the receptors are blocked, and as with most drugs, the bigger the dose, the bigger, and more rapid the effects, for both the therapeutic and the adverse side effects. (iii) Failure of therapeutic response may be because the dose is not enough. All these suppositions can now be challenged, but their legacy endures sometimes in routine prescribing practice.

A further important but little-explored area is the definition of individual optimal doses for the beneficial effects of these drugs. Guidelines for dosage, and dose equivalences between different drugs have been based on group averages derived from dose-finding trials. If there are large differences in individual sensitivity, this must mean that some patients are treated with doses far exceeding their optimal dose. Apart from acute motor side effects, antipsychotic drugs have serious long-term side effects. With the original neuroleptic drugs tardive dyskinesia and related conditions emerge. With “second-generation”, or “atypical” antipsychotic drugs, these problems are less but not eliminated, and other problems (weight gain, and the “metabolic syndrome”) have caused increasing concern. In addition, especially for the lower potency agents in both the first- and second-generation families of antipsychotic drugs, sedation is a common side effect, with major impact on quality of life. In view of these problems, definition of the optimal or minimum-effective dose for each patient is an important, unresolved issue. Whether there is a sharp threshold dose (with no effect below this dose, and full effect above it), or a region in the dose-response curve where the clinical response gradually increases with dose, is a difficult question to resolve, discussed further in PART II, Sect. 3.

Taking together the above areas of unresolved debate, there is room for a much better theoretical account of the action of these drugs. The present article attempts to construct a framework to resolve these issues. This builds on the author’s earlier publications, including detailed theory of the phenomenology of psychosis and the process of recovery during antipsychotic drug therapy [24,25,100,102,103]. The focus here is on pharmacological receptor types and related functions involved in actions of various classes of antipsychotic drugs.

## 2. PSYCHOSIS AS AN EXAGGERATION OF THE REINFORCEMENT FUNCTION OF DOPAMINE

Full recovery from florid psychotic episodes may take weeks, or even months, to run its full course, although relevant receptors are blocked within hours [99,103]. The psychological processes occurring during this period, while not widely studied, have been described in various ways. Using the language of learning theory they have been conceived as an extinction-like process [8]. Clinical accounts refer to a reduction in “behavioural impact” and “preoccupation” with delusional beliefs [106]. Using psychodynamic terminology they have been described as “working through conflicts of belief” [99,100,103]. A key to understanding these features of antipsychotic therapy is that one role of the neurotransmitter dopamine is as a central “reinforcement” signal, inferred from earlier behavioural work on instrumental conditioning. This clue led to the proposal in 1976 that psychosis is an abnormal exaggeration of the reinforcement function of dopamine, as it applies to typically-human cognitive processes, especially those involved in acquiring beliefs [97,98].

These processes, seen as formally similar to instrumental learning as studied in experimental animals, generate distorted beliefs, whose motivational significance is exaggerated. Antipsychotic drugs were then envisaged to slow down the process of formation of such abnormal beliefs, without erasing those already laid down in long-term memory. By overall slowing of mental activity, normal processes of restitution can come into play. Patients then gradually come to realize which beliefs are trustworthy, and to disregard those which have been a symptom of an illness. These processes take weeks or months to reach completion. Full restitution is then permitted by (but is not a direct consequence of) the medication. Beninger [8] developed a broadly similar concept of psychotic states, approaching the subject from the perspective of instrumental learning theory. The concept of psychosis as an overactivity of the reinforcement function of dopamine is now becoming accepted. It has many implications for further theory-development (explored in this paper), a practical implication being that once the dose is big enough to start to be effective, the eventual benefit is as much a matter of duration of treatment as of dose.

## 3. CHALLENGES TO THE “SINGLE RECEPTOR” ACCOUNT ACTION OF ANTIPSYCHOTIC DRUGS

- With classical neuroleptic drugs, the beneficial effects and adverse motor side effects are closely associated, and could plausibly be attributed to actions at a single receptor class. However, by the early 1970s it was known that the two effects could be dissociated: Certain drugs (clozapine and thioridazine), were known to be effective antipsychotic agents, but had a low tendency to produce motor side effects [57,143]. Subsequent research at the Janssen laboratories, based on collating profiles of receptor binding, clinical effects and side effects for a variety of antipsychotic agents [85,86] led to the conclusion that antipsychotic agents which are relatively free from motor side effects often combine D2-blocking potency with antagonism at one of the serotonin receptors (identified as the 5HT2a receptor). This led to the modern improved “atypical” antipsychotic agents - the “serotonin-dopamine antagonists”, or “SDAs”. Clozapine (but not thioridazine) fits this profile to some extent [96] but it is now clear that clozapine, and (as argued later) probably also thioridazine owe their distinctive clinical properties to other aspects of their basic pharmacology.
- By the early 1990s PET technology had been developed to measure, *in vivo*, the proportion of various receptors occupied when patients received therapeutic doses of antipsychotic drugs. For most antipsychotic drugs (including most “atypicals”), 65-70% occupancy of dopamine D2 receptor was required to achieve therapeutic benefits [41,114,149,154]. For typical neuroleptics, higher occupancy (75-80%) was needed before gross motor side effects were produced [72,148]. The occupancy needed for a therapeutic response may be an overestimate, since rigorous procedures to establish “minimum effective doses” were not adopted in these studies. Nevertheless, this evidence is compatible with the “single receptor” hypothesis for antipsychotic drugs, which achieve different effects at the same receptor at different occupancy thresholds. However, at least two drugs do not fit this pattern. Clozapine [41,78], and (amongst the newer drugs) quetiapine [78,82], can be therapeutically effective with much lower occupancy (~40%) of the D2 receptor. It has been suggested [136] that the special actions of these drugs depend on rapid release from binding to the D2 receptor, but the theory underlying this concept has been criticized [117]. It has also been suggested [138] that quetiapine, which has a short biological half-life, actually does have transiently high occupancy, this being sufficient for therapeutic action. This idea is still *sub judice*. However, several other lines of evidence, presented later in this paper, indicate that quetiapine is unusual compared to other antipsychotic drugs, in ways compatible with a different mode of action.
  These discrepancies mean that receptors other than, or additional to the D2 receptor are important in antipsychotic therapy, for clozapine and perhaps for quetiapine. This conclusion is supported by the fact that, while classical D2-blocking neuroleptics increase blood prolactin levels, an effect also seen during therapy with some atypical antipsychotic agents, this is not found with clozapine or quetiapine [27,53,75,95].
- If the idea is accepted that psychosis represents an exaggeration of the reinforcement function of dopamine, there is a severe paradox: That reinforcement function, whether examined in behavioural terms [9,105] or in terms of the dopamine-mediated synaptic change underlying it [77] derives from the actions of dopamine at D1 receptors, or from the mechanisms of synthesis of cyclic adenosine monophosphate (cAMP) [10], produced by D1 receptors, and not (mainly) at D2 receptors; yet, for the antipsychotic effects of neuroleptic drugs, the focus has always been on the D2 receptor, affinity for which scales with clinical potency of these drugs [30,137]. It has been claimed on the basis of several clinical trials [33,34,73,74] that D1 antagonists lack antipsychotic effects. These clinical trials are not rigorous refutation of the possibility that D1 antagonists have antipsychotic effects, mainly because, in those trials, an insufficient number of patients survived a long enough test period (at least 28 days) to draw definite conclusions [102,103,App.4]). A sharp paradox remains, if psychosis is viewed as a pathology of the reinforcement function of dopamine.

## 4. THEORY: THE INDIRECT MODE OF THERAPEUTIC ACTION OF D2-BLOCKING ANTIPSYCHOTIC DRUGS

To resolve this paradox, it has been proposed in several publications [101,102,103,105] that the D2-blocking agents act indirectly, the final common target of antipsychotic drugs of all classes being the D1 receptor, or the mechanism of synthesis of cAMP controlled by this receptor. This proposal fits evidence [94] that conditioned avoidance learning in rats (a test which is the best behavioural predictor of potency of antipsychotic agents in humans) can be attenuated in proportion to the affinity for either D1 receptors (for drugs which have higher potency at D1 than at D2 receptors) or at D2 receptors (for drugs whose potency is higher at D2 than at D1 receptors).

What steps could intervene between D2 blockade, and reduced D1 receptor activation (or reduced cAMP synthesis)? Two hypotheses are depicted in Fig. (**1**). In early formulations [101,105], it was proposed that *in vivo*, in free moving preparations, D2 blocking agents slowed the firing of midbrain dopamine neurones, whose dopamine release in the striatum was then reduced, “unloading” critical D1 receptors of their transmitter. It *was* known that D2 antagonists *increased* the firing rate of dopamine neurones, but that finding was based almost entirely on experiments in anaesthetized animals. However, single unit recording of dopamine neurones in free-moving rats, challenged with cataleptic doses of neuroleptic drugs, also showed increased firing, just as in anaesthetized preparations [B.I.Hyland, [pers com; 102: [Fig. (**2**)]). This is a decisive refutation of the original hypothesis.

More recently, new data have emerged, which provide an alternative, and better account of the stages intervening between D2 blockade and antipsychotic effects. In the striatum, a small proportion (2-3%) of neurones are “large cholinergic interneurones” [35]. Their neural activity, and release of acetyl choline (ACh) is controlled (*inter alia*) by dopamine, which inhibits these neurones at D2 receptors [55,84,135, 144,145,156]. Hence D2-blocking agents increase the firing rate, and release of ACh by these neurones. This is relevant to the motor side effects of antipsychotic agents, because these effects can be alleviated by anticholinergic agents. What are the actions at the cellular level, and the relevant receptors for this tonically-released ACh? There are at least five muscarinic receptors at which ACh might produce its actions [14,15] several of which (M1, M2, M4) are found in the striatum. Ultrastructural studies with special labelling methods [56,65] show that two of these (“M1” and “M4” receptors) are located on dendritic membranes of the principal neurones of the striatum, the medium spiny cells, a likely cellular target for production of both antipsychotic main effects and motor side effects of these drugs. It has been difficult to be specific about the cellular effect of these receptors, because few cholinergic agents are specific for one or other of these receptor types. Nevertheless from studies using gene-knockout techniques in mice, cell types in which specific receptors have been expressed, or, most recently, selective neurotoxins, insights into their different roles are appearing.

From gene knockout mice, it is known that M2-receptor-deficient mice no longer show tremor after administration of a cholinergic agonist [48]. However, this receptor type is located mainly on axon terminals of striatal neurones other than the medium-spiny type [56], probably including the cholinergic interneurones. The behavioural effects are therefore probably due to reduced ACh release in the striatum, rather than a receptor-specific postsynaptic effect of absence of this receptor type. The M1 receptor is located on medium spiny neurones, especially within their dendritic spines [56], and is present in almost all such neurones [158]. This receptor is known to suppress a potassium current in these neurones [141], leading to increased neuronal excitability. Since there are no specific agonists for this receptor it has not yet been possible to show directly the behavioural effects of stimulation of this receptor. However, it is known that in free-moving rats, many [20,79,110], perhaps all [102] medium spiny neurones increase their firing rates in low-dopamine states, characterised behaviourally by akinesia. This is a likely effect of reduction of dendritic potassium currents. Although never tested, the same increase probably occurs when akinesia and catalepsy are induced by D2-blocking agents. Therefore it is probable that the motor side effects of D2-blocking agents are mediated by increased cholinergic stimulation of M1 receptors on medium spiny neurones. The M4 receptor appears to limit cAMP synthesis, so that, when this receptor is activated, cAMP synthesis, stimulated in some other way, is decreased [115,116,118,132]. This makes M4 agonists similar in their effects on cAMP synthesis to D1 antagonists. An agent with putative M4 agonist actions, developed by Lilly has been tested and shown to have predicted neurochemical effects [125]. This similarity is supported by results of animal behavioural tests predictive of antipsychotic potency in humans [1,140]. It is then predicted that in animals treated with M4 agonists, processes of synaptic change, thought to underlie psychosis, would be retarded. It is proposed here (also [102,103]) that D2-blocking antipsychotic drugs, by accelerating firing in the cholinergic interneurones, increase ACh release which acts at M1 receptors, leading to motor side effects, and at M4 receptors to reduce dopamine-mediated synaptic potentiation In humans, the latter action sets in process the chain of events leading to alleviation of psychotic symptoms.

Other evidence supports this reasoning. Cholinergic interneurones are involved in one form of synaptic change in the striatum - long-term synaptic depression [153]. In this case the M1 muscarinic receptor is implicated. It is predicted that cholinergic mechanisms are also involved in another form of synaptic change - long-term synaptic potentiation - which is more relevant in the context of psychosis, and that the M4 receptor should be primarily involved. In monkeys, the acetyl cholinesterase inhibitor gallantamine inhibits psychosis-like behaviour induced by d-amphetamine [2]. Older evidence shows that anticholinesterases, or cholinomimetics may be helpful in psychotic states [67,120]. New data, based on the traditional practice in many Pacific islands of betel-nut chewing, suggest that actions of cholinergic alkaloids in betel nuts may also be antipsychotic [146]. Exactly which cholinergic receptor is implicated in cholinergic actions against psychosis, and whether any presently-available antipsychotic drugs (including the atypical ones and the unique drug clozapine) act in this way, is discussed below.

## 5. NEUROLEPTIC-RESISTANT PSYCHOSIS, AND INDIVIDUAL DIFFERENCES IN NEUROLEPTIC SENSITIVITY

It has long been suspected that some psychotic patients do not respond to standard antipsychotic drugs, but that there is something special about the drug clozapine in this respect. In 1988 [70] it was proved conclusively that clozapine *was* therapeutically effective, when other antipsychotic medications had failed. Since then, patients who are unresponsive to standard medications have increasingly been regarded as a separate class as far as drug treatment goes. Rigorous criteria, based on effects of previous drug treatment, are now adopted to define this class of patient.

Related to this is the suspicion that the patients who *do* respond to standard medications vary greatly, one from another in their sensitivity to the therapeutic effects of the drugs used. In the earliest days of neuroleptic treatment the German psychiatrist-neurologist H.-J.Haase, well aware of the unpleasant nature of the motor side effects, proposed that the dose of a neuroleptic which produced the least detectable signs of parkinsonism was the dose which produced all, or almost all of the therapeutic benefits [49,50]. To assess what he called the “neuroleptic threshold dose”, he developed a handwriting test, not itself a quantitative measure, although the estimates of individual thresholds obtained with this method *were* quantitative. Admittedly, with some drugs, therapeutic benefits can be achieved without motor side effects (see above), and the “neuroleptic threshold” concept also received criticism [142], as applied to classical neuroleptics. Nevertheless the concept gained general support from Angus and Simpson [5], authors with experience in both psychiatric and neurological evaluation. A later review of Haase’s handwriting test as a guide to neuroleptic doses including new data [83], offered some criticisms, but supported Haase’s concept in most cases. McEvoy and co-workers [92] conducted a rigorous test of Haase’s “threshold” concept, using another sensitive clinical test of motor function, also generally supporting the concept.

From such studies it was clear that the oral dose of a neuroleptic needed to achieve the “threshold” in the handwriting test varied greatly between patients. For the drug fluphenazine, the range of 2-14 mg/day (mean 4.56 mg/day) was reported [142], and from Haase’s own work, the means and standard deviations (SD) are 4.7±2.7 mg/day for the drug bromperidol [51], and 19.0±8.85 mg/day for the drug droperidol [52]. In the study of McEvoy and others [92] for the drug haloperidol, the threshold dose ranged, across 106 patients from <1 mg/d to 10 mg/day (mean 3.7±2.3), and was larger for patients previously treated with neuroleptics (4.3±2.4 mg/day) than for those receiving them for the first time (2.1±1.1 mg/day), a point of relevance in Sect. 7 (below). The “coefficients of variation” (CV) for the neuroleptic threshold in these studies is large (45-55%, or up to 62% in McEvoy’s study).

Direct assessment of variation in individual sensitivity to antipsychotic effects of these drugs is difficult. Outside psychiatry, individual sensitivity to many drugs is assessed by trying different doses in the same patient. However, treatment of *acute* psychotic episodes is often an emergency situation, not to be repeated if possible. Therefore, most dose-finding clinical trials average results across patients, with loss of data about individual sensitivity. A new approach to identifying individual drug sensitivity in treating acute psychosis is discussed in PART II (Sect. 3). Individual variation in minimum dose required in relapse-free *maintenance* treatment is also difficult to assess, because, in typical cases, relapse does not occur immediately on withdrawal or on dose reduction, but in a probabilistic fashion, sometime in the next year or two [39,54,58,60,69,90,111]. This principle also applies to atypical antipsychotic drugs [47,157]. Therefore study design generally has involved large groups of patients, with results expressed as percentage surviving without relapse as a function of time after the change. Again data on individual sensitivity to the relapse-prevention effects of the drug are hidden amongst the group data. The only viable approach is therefore to undertake careful longitudinal studies of each patient. A recent paper [66] did just that, not for typical psychosis, but in mentally-retarded adults, in relation to aggression and self-injurious behaviours. For haloperidol, across 16 patients, the minimum dose needed to prevent such behaviours (below which they were known to have occurred, and above which stable maintenance was possible) ranged from 0.5-18 mg/day (6.38±6.1 mg/day); for 4 patients on chlorpromazine, it ranged from 50-400 mg/day (200±147 mg/d); and for 11 patients on thioridazine, it ranged from 40-250 mg/day (131±65 mg/d). CVs ranged from ~50 to ~95%.

It might be suggested that individual variation in response, and even non-responsiveness, depends on differences in pharmacokinetics (absorption, protein binding, metabolism etc) of the drug. However, blood levels of medications in refractory patients are within, or (often) well above the range normally found to be effective [87]. For more typical patients, who do respond to medication, the plasma concentration of haloperidol needed to reach the “neuroleptic threshold”, showed a spread of values (4.9±2.9 ng/ml; CV=59%) just as wide as that for oral doses [91]. (In such studies haloperidol is the preferred drug, because its pharmacokinetics are simplest, with probably no active metabolites [44]).

Most neuroleptic drugs are extensively bound to plasma proteins, only unbound drug having access to the brain. If protein binding varies between subjects, “plasma level” would be an inaccurate guide to the effective concentration, to which the brain was exposed. One study [129] estimates, for 14 subjects, the fraction of haloperidol bound to plasma protein as 12.5±4.3%. Therefore, any measured plasma level of this drug would give a serum level subject to a CV of 34%. This is less than values cited above, for the CV of the “neuroleptic threshold”. Plasma levels have also been used to predict occupancy of D2 receptors [43]. If inter-subject variation in the fraction of free drug in the serum played a part in determining individual sensitivity, it would limit accuracy of prediction of occupancy of brain D2 receptors. However, using a simple model to make predictions, there was a mean error of only 6.6% (95% CI: 4.28-8.98%). Assuming occupancy to be determined by level of free drug in serum, the value given for its inter-subject variance [129] would lead to much larger error than this. One must conclude that variance in protein binding is unimportant in determining individual sensitivity. There must be an additional, substantial source of variance, more central than the serum levels of free drug.

What could this uncontrolled variable be? One possibility is the number or occupancy of dopamine D2 receptors in the striatum. Admittedly, if it is assumed that therapeutic effects depend directly on action at their target receptor, it is difficult to account for the wide (>ten-fold) range of individual sensitivities, and, in some cases, total non-responsiveness. Many studies compare D2 receptor numbers between normal subjects and those with schizophrenia, but only one [155] documents the relationship between receptor numbers and neuroleptic responsiveness, the mean number being 25-30% lower in the non-responsive than in the responsive cases. “Receptor number” is however a dynamic variable, subject to compensatory change, unless receptor loss is a consequence of cell loss; and there are few precedents for a disorder caused primarily by lasting excess or deficit of any receptor type, independent of cell loss. Two studies report on D2 receptor occupancy by antipsychotic drugs and the corresponding clinical response [72,113]. Patients with the same D2 occupancy showed very different degrees of clinical response. In another study [12] no correlation was found between occupancy and clinical improvement. Admittedly, the number of subjects in these studies was not large, and the measure of clinical response (the “vertical axis” in their plots) is not very exact, due to the difficulties of psychiatric rating scales. In refractory psychoses, classical neuroleptic drugs fail to produce a response even when D2 occupancy is at, or well above the levels known to be sufficient in more typical cases [28,46,122]. Although there is a possible confound in some of this evidence (see PART II, Sect. 4), *these results suggest that non-responsiveness of such patients has a basis in pharmacodynamics closer to the ultimate site of action than the D2 receptors*, and not in pharmacokinetics. This is compatible with the hypothesis that therapeutic action is an *indirect* consequence of blocking the dopamine D2 receptors (Sect. 4, above), the intervening stages being highly non-linear.

Estimates of threshold dose, or of minimum maintenance dose, though variable across subjects, are generally less than previously-recommended prescription guidelines based on group averages, implying that these doses are too large. This view gains support from studies of atypical antipsychotic drugs. One of these [45] used data showing that occupancy by dopamine of D2 receptors was about ~8.8% in normal subjects, but higher (~15.8%) in schizophrenia patients in a psychotic state, due to elevated dopamine release. To reduce occupancy by dopamine to normal levels, it was computed that antipsychotic drugs should give an occupancy of ~48%. This is substantially lower than the 65%, said to be needed for therapeutic action, which would give an occupancy by dopamine of only 7%. It is however very similar to the occupancy found empirically with doses of 10 mg/d of olanzapine [12]. The computed occupancy by dopamine would be 7.8% for olanzapine at 10 mg/d, and 5.5% for risperidone at 6 mg/d. If occupancy by the D2 blocker reached 80% (the level where major motor side effects appear), dopamine would then occupy only ~3% of receptor sites.

## 6. NUMBER OF STRIATAL CHOLINERGIC INTERNEURONES AS DETERMINANTS OF INDIVIDUAL SENSITIVITY AND NON-RESPONSIVENESS TO ANTIPSYCHOTIC DRUGS

From the foregoing discussion of striatal cholinergic mechanisms, the uncontrolled variable determining neuroleptic responsiveness could be the number of striatal cholinergic interneurones, or, equivalently, the amount of ACh which can be released when dopaminergic inhibition of these interneurones is blocked by D2 antagonists. This variable could determine individual sensitivity to antipsychotic agents, and, in the extreme case where there are very few such neurones, could lead to complete absence of a therapeutic response with classical antipsychotic agents.

Data from a few recent studies report on the numerical density of striatal cholinergic interneurones in post-mortem brain tissue. Two studies [61,62] compare densities between control subjects and those with schizophrenia. In various parts of the striatum, the CV across normal subjects ranged from 15-29% in the earlier study, and from 54-72% in the later one. These are large inter-subject variations, and, if the preceding reasoning holds true, would have an impact on individual sensitivity to actions of antipsychotic drugs. Data from animals support the argument: In different strains of mice, sensitivity to neuroleptic-induced motor effects (as measured by the ED50% for neuroleptic drugs to produce “catalepsy”) varies by a factor of more then ten [123]. The least-responsive strains, while having a number of neurochemical differences from the most-responsive strains, notably have 25-30% fewer striatal cholinergic neurones than the latter [31,59].

Another indication of loss of cholinergic neurones is reduction in the numbers of dopamine D2 receptors, since such receptors are located on these neurones. It has been shown in rats that regional levels of acetylcholinesterase [89] or counts of striatal cholinergic neurones [68] correlate with D2 receptor numbers. In cytological studies using the light microscope, these neurones *do* label for D2-selective ligands [7], the labelling being more prominent than for the more numerous principal cells of the striatum [16]. One might then expect that the least-responsive mouse strains would have lower striatal D2 receptors numbers than the most responsive strains. This was reported in one study [123] for a subregion of the caudate-putamen, but is not always seen [71]. D2 receptors in the striatum are found with locations on cellular elements additional to the cholinergic interneurones, including the spines of principal neurones [139] and dopaminergic terminals. From studies using cholinergic neurotoxins, no more than 50% of D2 receptors are lost if cholinergic cells are eliminated [32,160]. There are no reliable data on the proportion of striatal D2 receptors located presynaptically on dopaminergic terminals. After dopamine denervation of the striatum, a fall in D2 receptor numbers has been reported but this is not irreversible [136]. After a kainate lesion of the striatum, destroying cells but not axons, 35% of the D2 receptors remain, this deficit being apparently irreversible. The remaining receptors may then represent those on dopaminergic nerve terminals. Clearly there are complications which may prevent the prediction about D2 receptor number and cholinergic cell loss from being verified. Nevertheless, the inter-strain variation in sensitivity seen in mice is similar to that seen between individuals in humans. A prediction for human patients, with some empirical support [155], is that lower striatal D2 receptor numbers are associated with relative or absolute neuroleptic non-responsiveness for therapy of psychotic states.

## 7. “NEUROLEPTIC-INDUCED SUPERSENSITIVITY PSYCHOSIS”

In 1978 Chouinard and collaborators [23,26] suggested that, in some patients treated with neuroleptic drugs, the dose needed to control psychotic symptoms gradually rose over months and years of treatment, so that symptoms could break through, despite a previously-adequate dose, or could appear quickly if attempts were made to reduce the dose. In the most severe cases [22], the classical neuroleptic drugs lost their effectiveness completely, although patients might still respond well to clozapine or other non-standard medications. Initially [26] it was proposed that this syndrome was a consequence of changes in receptor numbers. However, in 1993 [104] it was argued that the more fundamental change was a reduction in the number of striatal cholinergic interneurones. The proposed pathological process was that antipsychotic drugs might, in some patients, increase neural activity in these neurones so much that they became vulnerable to cytotoxic processes. The exact mechanisms of this are beyond the scope of this article, but it was thought probable that they reflect a general vulnerability to neuronal damage, characteristic of some people, independent of their risk of psychotic illnesses. From evidence and reasoning presented earlier in this paper, one might then expect that, in the study of McEvoy and co-workers [92], the higher mean dose to achieve the neuroleptic threshold in previously-treated, compared to neuroleptic-naive patients may reflect neuroleptic-induced reduction in numbers of cholinergic interneurones.

Since then, it has been documented that, in brains from persons with schizophrenia, striatal cholinergic interneurones *are* reduced in numbers, compared to control subjects [61,62]. These studies did not link the reduction specifically to a previous history of neuroleptic treatment, or to neuroleptic non-responsiveness, but all the patients *were* treated extensively with antipsychotic drugs before death. Though not proven, it is therefore plausible to suggest that the loss of cholinergic neurones was a consequence of the neuroleptic treatment. However, it should also be made clear that some patients with psychotic illnesses are refractory to treatment with neuroleptic drugs, right from the start of their illness. Thus, non-responsiveness to neuroleptics, and reduced numbers of striatal cholinergic interneurones cannot be attributed solely to the effect of antipsychotic drugs: Other patients probably have these abnormalities *ab initio*.

## 8. CHOLINERGIC INTERNEURONES AS “NEURODYNAMIC STABILIZERS”

What is the real function of the cholinergic interneurones? Consider the design of neural machinery for acquiring neural representations controlling motivationally-favourable executive decisions [102]. Hypothetically, this machinery is realized in the striatum, associated parts of the basal ganglia, and the other pathways by which those functions might be expressed. The most basic framework for this machine consists of the principle neurones of the striatum, their input and output pathways, and the dopaminergic reinforcement system. Why, then, should it also be necessary to include within the striatum the small but influential fraction of cholinergic interneurones? In the book “A theory of the basal ganglia and their disorders” [102], a tentative answer was provided to this question. In the dynamic relation between the striatum, other parts of the basal ganglia, the “motor thalamus” and the cerebral cortex, there is a very numerous set of recursive connectional loops. Outputs from this system, such as signals controlling behaviour with “favourable consequences”, or its equivalent for humans at the level of internalised thoughts, is capable of controlling the dopaminergic reinforcement system, which operates by strengthening critical afferent striatal synapses in these recursive loops. There is then the potential for uncontrolled positive feedback, with pathological consequences at neurodynamic and psychological/behavioural levels. The symptoms of such pathology could be psychosis (in the cognitive domain), or abnormal involuntary movements - dyskinesias - (in the motor domain). (Dyskinesias were referred to as “psychosis of movement” [102]). However, neither psychosis nor dyskinesias occur commonly in usual circumstances when the dopaminergic system is activated. Psychosis, when it does occur in humans, has some other precipitating factor, such as stimulant drugs (which push the levels of dopamine activity well beyond their normal limit), or other endogenous illnesses (such as schizophrenia or bipolar disorder) which have many defining features more fundamental than dopamine-mediated psychosis. Likewise, dyskinesias are not part of the normal motor repertoire, even when large reinforcements are given in a normal fashion. They may occur, however, when dopaminergic tone is pushed to extreme levels by drugs [107, 108,131,133,134,152]. Similarly, in early Parkinson’s disease, dyskinesias are rare in response to treatment with L-DOPA and similar drugs, but emerge as a result of additional changes, in advanced stages of the disease [29,109], or restricted to the more severely affected side [63,76].

It was suggested by Miller and Chouinard [104], that L-DOPA-induced dyskinesias arise in Parkinson’s disease when, as a complication additional to loss of midbrain dopamine cells, there was progressive loss of cholinergic interneurones in the striatum. This was seen as a pathology in some ways parallel to that leading to tardive dyskinesia after prolonged neuroleptic treatment, or (in the cognitive domain) to the processes leading to neuroleptic-induced supersensitivity psychosis. There is indeed evidence for loss of cholinergic markers from the striatum in some cases of advanced Parkinson’s disease [88,112,126]. Thus, it appears that the integrity of the striatal cholinergic interneuronal function is somehow necessary for stable operation of the cortico-basal ganglionic system, without which uncontrolled positive feedback may occur.

A possible mechanism for this stabilizing role, involving striatal cholinergic neurones, and receptors already mentioned, was proposed by Miller [102]. At times when dopaminergic tone is high, strengthening of excitatory synaptic input to medium spiny cells is favoured by either increased D1 activation, or reduced muscarinic M4 activation, both contributing to increased cAMP formation within these cells. There is then a potential for positive feedback loops to develop between basal ganglia and cortex. However, as mentioned above, muscarinic M1 receptors located on striatal medium spiny cells control conductance in a potassium channel and also neuronal excitability. Activation of these receptors is probably the mechanism leading to Parkinsonian symptoms in low-dopamine states, and in the opposite sense, explains the action of anticholinergic drugs used to alleviate motor side effects of neuroleptic drugs. When dopaminergic tone is elevated, cholinergic activation of the M1 receptors is reduced, potassium conductance increases, and neuronal excitability is lowered. This has several beneficial effects: It reduces the tendency to motor side effects; it increases the signal-to-noise ratio of the impulse traffic in the medium spiny neurones; and it also reduces the tendency to development of unwanted positive feedback loops, manifest as psychosis or dyskinesia. That dyskinesias are nevertheless mediated by extreme reduction of cholinergic tone is shown by the fact that they can be alleviated using anticholinesterases [19]. At least one can say that the range of stable operation, from low to high levels of dopamine tone, is increased, compared to that obtaining in the basic cellular framework of the striatum where there are no cholinergic neurones. However, *with* these neurones, stable function of the cortico-basal ganglionic system can occur without problems, in a much wider set of circumstances than in the basic framework by itself.

Even with a normal compliment of cholinergic interneurones, animals and humans may sometimes display pathologies. Rat strains are known which can display catalepsy, even with no pharmacological challenge [81], which is then exacerbated by cholinergic drugs, and reduced by atropine. While its neural basis in not well studied, it appears to involve the striatum [80,119]. The vulnerability to this dysfunction appears to have, in part, a genetic basis [93], and it would be interesting to know if this trait is associated with heightened sensitivity to neuroleptic-induced catalepsy, or increased level of striatal cholinergic markers or cholinergic interneurones. At the other extreme, in humans, underlying abnormalities elsewhere in the nervous system [103], can lead to such excesses of dopaminergic tone that episodes of florid psychosis occur, again with no pharmacological trigger, as in schizophrenia.

This reasoning leads to a prediction: If cholinergic neurones are lost, there will be an enhanced tendency to an unusual form of psychosis, or to dyskinesia, in high-dopamine states. Evidence mentioned above fits this expectation, with regard to dyskinesias. In Parkinson’s disease, treatment with dopamine agonists does not usually lead to psychosis, but may do so more commonly as the disease advances [42]. These pathologies should not be controllable by D2 antagonists (whose efficacy has now been lost or reduced, along with the cholinergic interneurones), but will be controllable by drugs which act more directly on the final common target for antipsychotic drugs of all classes, namely the D1 dopamine receptor or the M4 muscarinic receptor. Are there any such drugs?

## 9. ACTIONS OF CLOZAPINE

Table **1** shows, for various antipsychotic drugs, affinities (Ki’s) for the D1 and D2 dopamine receptors, and for the M1 and M4 muscarinic receptors, as well as ED50% values for these drugs as M4 agonists. Most antipsychotic drugs (not tabulated) have much higher affinities for D2 than D1 receptors, but for some (clozapine, thioridazine, fluperlapine, chlorprothixene) the respective affinities are not very different, and for a few (clozapine, one of the enantiomers of thio-ridazine, fluperlapine), affinities for the D1 receptor are somewhat higher than those for the D2 receptor. Potentially then, these drugs might owe their action to D1 antagonism, or to intracellular consequences of this, namely decreased cAMP synthesis, upon which dopamine-mediated synaptic change depends. However the data on affinities for M4 receptors lead to another conclusion: For clozapine, the affinity for the M4 receptor is higher than for either D1 or D2 receptors. Clozapine appears to be an agonist, or a partial agonist at M4 receptors [18,159], but not a pure antagonist. Consequently, it can be proposed that the distinctive action of clozapine is based on its stimulation of M4 receptors, leading to reduction of cAMP formation, and reduction of dopamine-mediated synaptic potentiation, and, at the psychological or behavioural level, reduction of reinforcement processes. Since it acts more directly on the ultimate target than the D2-blocking antipsychotic drugs, it does not require the mediating stage of the cholinergic interneurones. It is therefore effective in those cases where, either *ab initio*, or as a result of prior neuroleptic treatment, these neurones are reduced in number, and there is insufficient capacity for ACh release to activate M4 receptors (and limit cAMP production, as the usual response to D2-blocking antipsychotic drugs). The reason why clozapine has antipsychotic effects, but does not cause motor side effects is then that its pattern of action at the different muscarinic receptors on medium spiny neurones is different from that of endogenously-released acetyl choline. Likewise, anticholinergic drugs can be used to reduce motor side effects without stopping the therapeutic effect of classical neuroleptic drugs because they block actions of endogenous ACh at M1 receptors, but not at M4 receptors. (Most such drugs have higher affinity at M1 than at M4 muscarinic receptors [13]).

In Sect. 8 (above), it was suggested that drugs which directly reduce activation of the substrate for synaptic potentiation in the striatum should be effective in limiting both dyskinesias and psychosis triggered by dopamine agonists, in advanced stages of Parkinson’s disease. Clozapine fulfils both these predictions: It alleviates L-DOPA-induced dyskinesias, without exacerbating the underlying condition, in regular treatment [11], under test conditions (apomorphine challenge: [36]), and in a randomised double-blind trial [37]. From reasoning developed here, this would be expected of a drug with M4 agonist, and M1 antagonist properties. The decline of symptoms is progressive over several weeks [121], similar to that achieved with clozapine for psychosis, supporting the model developed here. When L-DOPA or similar drugs produce psychosis in Parkinson’s disease, clozapine is also effective treatment (see: review of ~200 cases [6]; also [40,124,151]) without exacerbating parkinsonian symptoms. This differential action, not seen with other atypical antipsychotics [38] depends on use of low doses, although the dose needed to alleviate psychosis depends on its severity [130]. Presumably at higher doses, the D2-blocking potential of clozapine comes into play, thereby inducing remaining cholinergic interneurones to increase ACh release, and exacerbate parkinsonian symptoms. As with therapy for psychoses of other origins, and L-DOPA-induced dyskinesias, improvement takes some weeks to reach completion [150]. It has also been noted that for some patients, even the cardinal symptoms of Parkinson’s disease may benefit from clozapine [6], a result which might arise from direct antagonism at muscarinic M1 receptors. *(continued, in PART II).*

## Figures and Tables

**Fig. (1):**
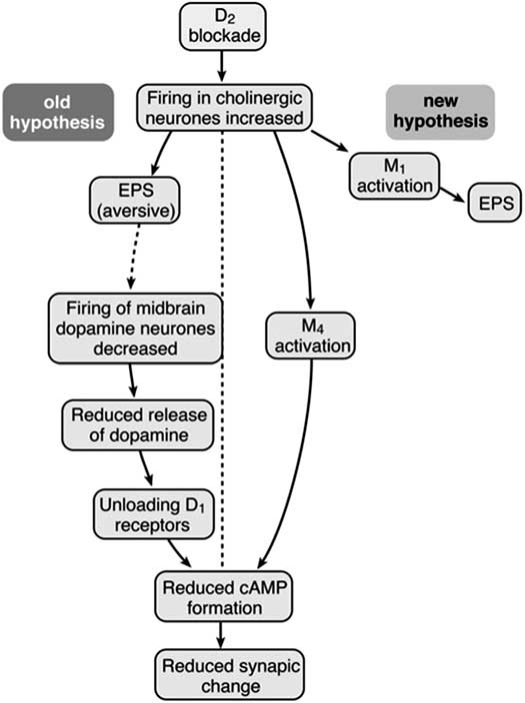
**Alternative hypotheses for indirect action of dopamine D2-receptor-blocking antipsychotic drugs.** Both hypotheses postulate increased firing of striatal cholinergic interneurons, and that the final common target of antipsychotic drugs is reduced formation of cAMP. *Left*: Indirect action mediated by the aversive effects of extrapyramidal side effects (EPS). *Right*: Indirect action mediated by increased activation of striatal muscarinic M4 receptors.

**Fig. (2):**
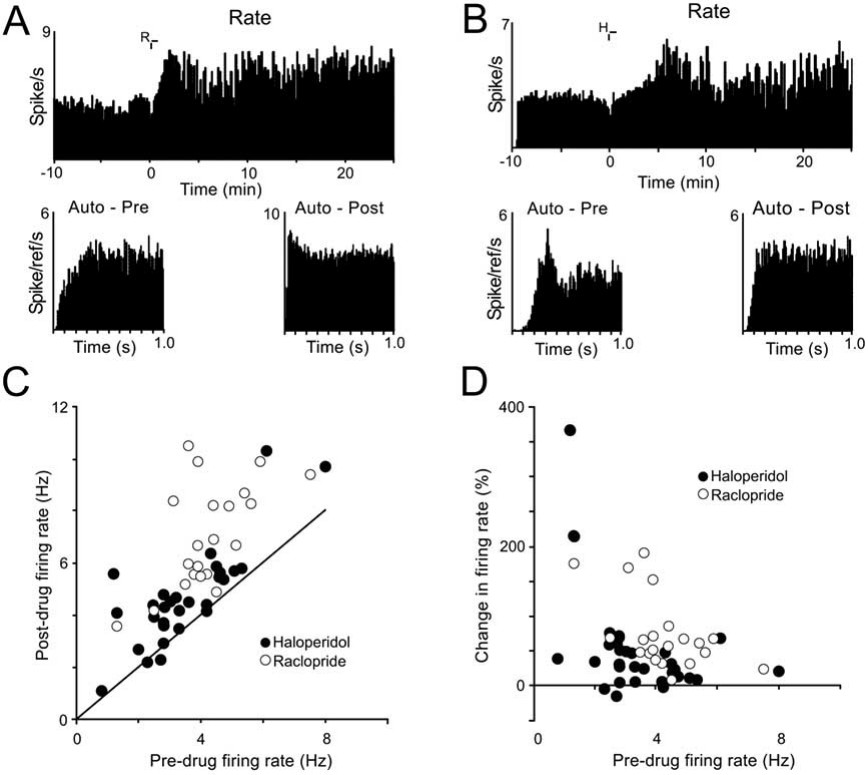
**Effect of D2-receptor blocking drugs on firing rate and firing pattern of dopamine cells recorded in free-moving rats. A:-** Rate-meter histogram (top panel; one-second bins) shows increased firing rate in this cell after raclopride injection at time 0 (“R”). The autocorrelogram plots below the histogram, calculated from the 10-minute pre-drug control period (left) and the period 10-20 minutes post-drug (right), show irregular firing before, and enhanced bursting after injection in this cell. **B:-** A cell showing increased firing rate and decreased regularity of firing after haloperidol (“H”). **C:-** Relationship between baseline (pre-drug) firing rate and post-drug firing rate, calculated from period 10-20 minutes post-injection. Diagonal line shows line of equality (no change). **D:-** Relationship between baseline firing rate and the change in firing rate (difference between pre- and post-drug firing rates expressed as a percentage of baseline rate) induced by D2 antagonists. (from B.I.Hyland, personal communication, with thanks).

**Table 1.** Affinities of some Atypical Antipsychotic Agents at D1 and D2 Dopamine Receptors, and at M1 and M4 Muscarinic Receptors

Drug	Affinity (Ki), or EC50% as an agonist (nM[ref])				
	D2	D1	M1	M4	
				Affinity	ED50% as Agonist [A]
Chlorprothixene	3.2[a]	16[a]	11[b]	18[b]	?
Clozapine	90[c],125[d]	55[c],85[d]	3.1[b],1.4[e]	11[b],6[e]	60[f]
Fluperlapine	245[k]	85[k]	8.8[b]	14[b]	600[f]
Mesoridazine [B]	5[g]	?	10[b]	19[b]	?
1	2.2[h]	103[h]	?	?	?
2	304[h]	1963[h]	?	?	?
3	426[h]	620[h]	?	?	?
4	1.2[h]	317[h]	?	?	?
Olanzapine	2[j],11[d]	10[j],31[d]	2.5[b]	10[e]	1900[f]
Quetiapine	770[i],160[d],69[j]	455[d], 390[j]	120[e]	660[e]	“almost no effect” [f]
Thioridazine [C]	9.5[c]	21[c]	2.7[b]	9[b,e]	[B][f]
+	4.68[l]	72.4[l]	?	?	?
-	12.7[l]	7.08[l]	?	?	?

Notes:

A: Reduction of forskolin-stimulated cAMP synthesis [159], in Chinese hamster ovary (CHO) cells, in which muscarinic M4 receptors were expressed.

B: see comments in PART II, Sect. 2.

C: Thioridazine is usually available in racemic form.

References:

a: [64]

b: [13]

c: [3]

d: [17]

e: [18]

f: [159]

g: [128]

h: [21]

i: [127]

j: [161]

k: [4]

l: [147].

## References

[1] Andersen MB, Fink-Jensen A, Peacock L, Gerlach J, Bymaster F, Lundbaeck JA, Werge (2003). The muscarinic M1/M4 receptor agonist xanomeline exhibits antipsychotic-like activity in Cebus apella monkeys. Neuropsychopharmacology.

[2] Andersen MB, Werge T, Fink-Jensen A (2007). The acetylcholinesterase inhibitor galantamine inhibits d-amphetamine-induced psychotic-like behavior in Cebus monkeys. J. Pharmacol. Exp. Ther.

[3] Andersen PH, Gronvald FC, Jansen JA (1985). A comparison between dopamine-stimulated adenylate cyclase and 3H-SCH 23390 binding in rat striatum. Life Sci.

[4] Andersen PH (1988). Comparison of the pharmacological characteristics of [3H] raclopride and [3H]SCH23390 binding to dopamine receptors *in vivo* in mouse brain. Eur. J. Pharmacol.

[5] Angus JWS, Simpson GM (1970). Handwriting changes and response to drugs: A controlled study. Acta Psychiatr. Scand. Suppl.

[6] Anzou P, Özsancak C, Hannquin D, Moore N, Augustin P (1986). Clozapine for the treatment of psychosis in Parkinson's disease. Acta Neurol. Scand.

[7] Ariano MA, Kang HC, Haugland RP, Sibley DR (1991). Multiple fluorescent ligands for dopamine receptors. II: Visualization in neural tissues. Brain Res.

[8] Beninger RJ (1983). The role of dopamine in locomotor behavior and learning. Brain Res. Brain Res. Rev.

[9] Beninger RJ, Miller R (1998). Dopamine D1-like receptors and reward-related incentive learning. Neurosci. Biobehav. Rev.

[10] Beninger RJ, Nakonechny PL, Todd MJ (1996). Inhibition of protein kinase A in the nucleus accumbens blocks amphetamine-produced conditioned place preference in rats. Abstr. Soc. Neurosci.

[11] Bennett JP, Landow ER, Dietrich S, Schuh LA (1994). Suppression of dyskinesias in advanced Parkinson's disease: moderate daily clozapine doses provide long-term dyskinesia reduction. Mov. Disord.

[12] Bernardo M, Parellada E, Lomeña F, Catafau AM, Font M, Gómez JC, López-Carrero C, Gutiérrez F, Pavla J, Salamero M (2001). Double-blind olanzapine *vs*. haloperidol D2 dopamine receptor blockade in schizophrenic patients: a baseline-endpoint [I123]IBZM SPECT study. Psychiatry Res.

[13] Bolden C, Cusack B, Richelson E (1992). Antagonism by anti-muscarinic and neuroleptic compounds at the five cloned human muscarinic cholinergic receptors expressed in Chinese hamster ovary cells. J. Pharmacol. Exp. Ther.

[14] Bonner TI, Buckley NJ, Young AC, Brann MR (1987). Identification of a family of muscarinic acetylcholine receptor genes. Science.

[15] Bonner TI, Young AC, Brann MR, Buckley NJ (1988). Cloning and expression of the human and rat m5 muscarinic acetylcholine receptor gene. Neuron.

[16] Brené S, Lindefors N, Herrera-Marschitz M, Persson H (1990). Expression of dopamine D2 receptor and choline acetyltransferase mRNA in the dopamine deafferented rat caudate-putamen. Exp. Brain Res.

[17] Bymaster FP, Calligaro DO, Falcone JF, Marsh RD, Moore NA, Tye NC, Seeman P, Wong DT (1996). Radioreceptor binding profile of the atypical antipsychotic olanzapine. Neuropsychopharmacology.

[18] Bymaster FP, Felder CC, Tzavara E, Nomikos GG, Calligaro DO, Mckinzie DL (2003). Muscarinic mechanisms of antipsychotic atypicality. Prog. Neuropsychopharmacol. Biol. Psychiatry.

[19] Caroff SN, Martine R, Kleiner-Fisman G, Eisa M, Lorry A, Gallop R, Stern MB, Duda JE (2006). Treatment of levodopa-induced dyskinesias with donezepil. Parkinsonism Relat. Disord.

[20] Chen MT, Morales M, Woodward DJ, Hoffer BJ, Janak PH (2001). *In vivo* extracellular recording of striatal neurons in the awake rat following unilateral 6-hydroxydopamine lesions. Exp. Neurol.

[21] Choi S, Haggart D, Lawrence T, Cuny GD (2004). Synthesis, receptor binding and functional studies of mesoridazine stereoisomers. Bioorg. Med. Chem. Lett.

[22] Chouinard G (1990). Severe cases of neuroleptic-induced supersensitivity psychosis, diagnostic criteria for the disorder and its treatment. Schizophr. Res.

[23] Chouinard G, Jones BD (1980). Neuroleptic-induced supersensitivity psychosis: clinical and pharmacologic characteristics. Am. J. Psychiatry.

[24] Chouinard G, Miller R (1999a). A rating scale for psychotic symptoms (RSPS): Part I: theoretical principles and subscale 1: perception symptoms (illusions and hallucinations). Schizophr. Res.

[25] Chouinard G, Miller R (1999b). A rating scale for psychotic symptoms (RSPS): Part II: Subscale 2: distraction symptoms (catatonia and passivity experiences); subscale 3: delusions; and semistructured interview (SSCI-RSPS). Schizophr. Res.

[26] Chouinard G, Jones BD, Annable L (1978). Neuroleptic-induced supersensitivity psychosis. Am. J. Psychiatry.

[27] Copolov DL, Link CG, Kowalcyk BA (2000). A multicentre, double-blind, randomized comparison of quetiapine (ICI 204,636, 'Seroquel') and haloperidol in schizophrenia. Psychol. Med.

[28] Coppens HJ, Sloogg CJ, Paans AMJ, Wiegman T, Vaalburg W, Korf J (1991). High central D2-dopamine receptor occupancy as assessed with positron emission tomography in medicated but therapy-resistant schizophrenic patients. Biol. Psychiatry.

[29] Cotzias GC, Papavasiliou PS, Gellene R (1969). Modification of parkinsonism: chronic treatment with L-DOPA. N. Engl. J. Med.

[30] Creese I, Burt DR, Snyder SH (1976). Dopamine receptor binding predicts clinical and pharmacological potencies of anti-schizophrenic drugs. Science.

[31] Dains K, Hitzemann B, Hitzemann R (1996). Genetics, neuroleptic response and the organization of cholinergic neurons in the mouse striatum. J. Pharmacol. Exp. Ther.

[32] Dawson VL, Dawson TM, Filloux FM, Wamsley JK (1988). Evidence of dopamine D2 receptors on cholinergic interneurons in the rat caudate putamen. Life Sci.

[33] De Beaurepaire R, Labelle A, Naber D, Jones BD, Barnes TRE (1995). An open trial of the D1 antagonist SCH 39166 in six cases of acute psychotic states. Psychopharmacology (Berl).

[34] Den Boer JA, van Megen HJGM, Fleischhacker WW, Louwerens JW, Slaap BR, Westenberg HGM, Burrows GD, Srivasta ON (1995). Differential effects of the D1-DA receptor antagonist SCH 39166 on positive and negative symptoms of schizophrenia. Psychopharmacology (Berl).

[35] DiFiglia M (1987). Synaptic organization of cholinergic neurons in the monkey striatum. J. Comp. Neurol.

[36] Durif F, Vidailhet M, Assal F, Roche C, Bonnet AM, Agid Y (1997). Low-dose clozapine improves dyskinesias in Parkinson's disease. Neurolog.

[37] Durif F, Debilly B, Galitzky M, Morand D, Viallet F, Borg M, Thobois S, Broussolle E, Rascol O (2004). Clozapine improves dyskinesias in Parkinson disease: a double-blind placebo-controlled study. Neurology.

[38] Ellis T, Cudkowicz ME, Sexton PM, Growdon JH (2000). Clozapine and risperidone treatment of psychosis in Parkinson's disease. J. Neuropsychiatry Clin. Neurosci.

[39] Engelhardt DM, Rosen B, Freedman N, Margolis R (1967). Phenothiazines in prevention of psychiatric hospitalization - a re-evaluation. Arch. Gen. Psychiatry.

[40] Factor SA, Friedman JH, Lannon MC, Oakes D, Bourgeois K, Parkinson Study Group (2001). Clozapine for the treatment of drug-induced psychosis in Parkinson’s disease: results of the 12 week open label extension in the PSYCLOPS trial. Mov. Disord.

[41] Farde L, Nordström A-L, Wiesel F-A, Pauli S, Halldin C, Sedvall G (1992). Positron emission tomographic analysis of central D1 and D2 dopamine receptor occupancy in patients treated with classical neuroleptics and clozapine. Arch. Gen. Psychiatry.

[42] Fischer P, Danielczyk W, Simanyi M, Streifler MB (1990). Dopaminergic psychoses in advanced Parkinson's disease. Adv. Neurol.

[43] Fitzgerald PB, Kapur S, Remington G, Roy P, Zipursky RB (2000). Predicting haloperidol occupancy of central dopamine D2 receptors from plasma levels. Psychopharmacology (Berl).

[44] Forssman A, Ohman R (1976). Pharmacokinetic studies on haloperidol in man. Curr. Ther. Res.

[45] Frankle WG, Gil R, Hacket E, Mawlawi O, Zea-Ponce Y, Zhu Z, Kochan LD, Cangiano C, Slifstein M, Gorman JM, Laruelle M, Abi-Dargham A (2004). Occupancy of dopamine D2 receptors by the atypical antipsychotic drugs risperidone and olanzapine: theoretical implications. Psychopharmacology (Berl).

[46] Geaney DP, Ellis PM, Soper N, Shepstone BJ, Cowen PJ (1992). Single photon emission tomography assessment of cerebral dopamine D2 receptor blockade in schizophrenia. Biol. Psychiatry.

[47] Glick ID, Berg PH (2002). Time to study discontinuation, relapse and compliance with atypical or conventional antipsychotics in schizophrenia and related disorders. Int. Clin. Psychopharmacol.

[48] Gomeza J, Shannon H, Kostenis E, Felder C, Zhang L, Brodkin J, Grinberg A, Sheng H, Wess J (1999). Pronounced pharmacologic deficits in M2 muscarinic acetylcholine receptor knockout mice. Proc. Natl. Acad. Sci. USA.

[49] Haase H-J (1954). Über das Vorkommen und Deutung des psychomotorischen Parkinsonsyndroms bei Megaphen bzw. Largactil-Dauerbehandlung. Nervenarzt.

[50] Haase H-J, Haase H-J, Janssen PAJ (1965). Clinical Observations on the Actions of Neuroleptics. Actions of Neuroleptic Drugs: A Psychiatric, Neurological and Pharmacological Investigation.

[51] Haase H-J, Kaumeier S, Schwarz H, Gundei A, Linde OK, Maetz H, Scheel R, Stripf A, Stripf L (1978). Open study with bromperidol (C-C 2489), a new neuroleptic, for the determination of the neuroleptic threshold and the neuroleptic therapeutic range. Pharmakopsychiatry.

[52] Haase H-J, Kaumeier S, Schwarz S, Gundel A, Libde OK, Höness V R, Bomhard ER, Rauwolf ER, Schel R, Seyfferth H, Stripf A, Stripf L (1980). Clinical trial of droperidol for the determination of the neuroleptic threshold and the neuroleptic-therapeutic range. Int. Pharmacopsychiatry.

[53] Haddad PM, Wieck A (2004). Antipsychotic-induced hyperprolactinaemia: mechanisms, clinical features and management. Drugs.

[54] Heresco-Levy U, Greenburg D, Lerer B, Dasberg H, Brown WA (1993). Trial of maintenance neuroleptic dose reduction in schizophrenic outpatients: two-year outcome. J. Clin. Psychiatry.

[55] Herrting G, Zumstein A, Jakisch R, Hoffman R, Starke K (1980). Modulation by endogenous dopamine of the release of acetylcholine in caudate nucleus of the rabbit. Naunyn Schmiedebergs Arch. Pharmacol.

[56] Hersch SM, Gutekunst CA, Rees HD, Heilman CJ, Levey AI (1994). Distribution of m1-m4 muscarinic receptor proteins in the rat striatum: light and electron microscopic immunocytochemistry using subtype-specific antibodies. J. Neurosci.

[57] Hippius H, Sedvall G, Uvnas B, Zotterman Y (1976). On the Relations Between Antipsychotic and Extrapyramidal Effects of Psychoactive Drugs. Antipsychotic Drugs: Pharmacodynamics and Pharmacokinetics (Werner-Gren International Symposium Series, vol. 25).

[58] Hirsch S, Bowen J, Emami J, Cramer P, Jolley A, Haw C, Dickinson M (1996). A one-year prospective study of the effect of life events and medication in the aetiology of schizophrenic relapse. Br. J. Psychiatry.

[59] Hitzemann R, Qian Y, Hitzemann B (1993). Dopamine and acetylcholine cell density in the neuroleptic responsive (NR) and neuroleptic nonresponsive (NNR) lines of mice. J. Pharmacol. Exp. Ther.

[60] Hogarty GE, McEvoy JP, Munetz M, Barry ALD, Bartone P, Cather R, Cooley SJ, Urich RF, Carter M, Madonia MJ (1988). Dose of fluphenazine, familial expressed emotion, and outcome in schizophrenia. Arch. Gen. Psychiatry.

[61] Holt DJ, Herman MM, Hyde TM, Kleinman JE, Sinton CM, German DC, Hersh LB, Graybiel AM, Saper CB (1999). Evidence for a deficit in cholinergic interneurons in the striatum in schizophrenia. Neuroscienc.

[62] Holt DJ, Bachus SE, Hyde TE, Wittie M, Herman MM, Vangel M, Saper CB, Kleinman JE (2005). Reduced density of cholinergic interneurons in the ventral striatum in schizophrenia: an *in situ* hybridization study. Biol. Psychiatry.

[63] Horstink MWIM, Zijlmans JC, Pasman JW, Berger HJ, van't Hof MA (1990). Severity of Parkinson's disease is a risk factor for peak-dose dyskinesia. J. Neurol. Neurosurg. Psychiatry.

[64] Hyttel J (1983). SCH 23390 - The first selective dopamine D-1 antagonist. Eur. J. Pharmacol.

[65] Ince E, Ciliax BJ, Level AI (1997). Differential expression of D1 and D2 dopamine and M4 muscarinic acetylcholine receptor proteins in identified striatonigral neurons. Synapse.

[66] Janowsky DS (2005). Minimally effective doses of conventional antipsychotic medications used to treat aggression, and self-injurious behaviors in mentally retarded adults. J. Clin. Psychopharmacol.

[67] Janowsky DS, El-Yousef MK, Davis JM, Selerke HJ (1973). Antagonistic effects of physostigmine and methylphenidate in man. Am. J. Psychiatry.

[68] Joyce JN, Marshall JF (1985). Striatal topography of D2 receptors correlates with indexes of cholinergic neuron localization. Neurosci. Lett.

[69] Kane JM, Rifkin A, Woerner M, Reardon G, Sarantakos S, Schiebel D, Ramirez I (1983). Low-dose neuroleptic treatment of outpatient schizophrenics. I: Preliminary results for relapse rates. Arch. Gen. Psychiatry.

[70] Kane JM, Honigfeld G, Singer J, Meltzer H, Clozaril Collaborative Study Group (1988). Clozapine for the treatment-resistant schizophrenic. Arch. Gen. Psychiatry.

[71] Kanes SJ, Hitzemann BA, Hitzemann J (1993). On the relationship between D2 receptor density and neuroleptic-induced catalepsy among eight inbred strains of mice. J. Pharmacol. Exp. Ther.

[72] Kapur S, Zipursky R, Jones C, Remington G, Houle S (2000). Relationship between D(2) occupancy and side effects: a double-blind PET study of first-episode schizophrenia. Am. J. Psychiatry.

[73] Karle J, Clemmensen L, Hansen L, Andersen M, Andersen J, Fensbo C, Sloth-Nielsen M, Lublin H, Gerlach J (1995). NNC-01-0687, a selective dopamine receptor antagonist, in the treatment of schizophrenia. Psychopharmacology (Berl).

[74] Karlsson P, Smith L, Farde L, Harnryd C, Sedvall G, Wiesel FA (1995b). Lack of apparent antipsychotic effect of the D1-dopamine receptor antagonist SCH39166 in acutely ill schizophrenic patients. Psychopharmacology (Berl).

[75] Kasper S, Muller-Spahn F (2000). Review of quetiapine and its clinical applications in schizophrenia. Exp. Opin. Pharmacother.

[76] Kempster PA, Gibb WR, Stern GM, Lees AJ (1989). Asymmetry of substantia nigra neuronal loss in Parkinson's disease and its relevance to the mechanism of levodopa related motor fluctuations. J. Neurol. Neurosurg. Psychiatry.

[77] Kerr JN, Wickens JR (2001). Dopamine D-1/D-5 receptor activation is required for long-term potentiation in the rat neostriatum *in vitro*. J. Neurophysiol.

[78] Kessler RM, Ansari AS, Riccardi P, Li R, Jayathilake K, Dawant B, Meltzer HY (2006). Occupancy of striatal and extrastriatal dopamine D2 receptors by clozapine and quetiapine. Neuropsychopharmacology.

[79] Kish LJ, Palmer MR, Gerhardt GA (1999). Multiple single-unit recordings in the striatum of freely moving animals: effects of apomorphine and d-amphetamine in normal and unilateral 6-hydroxydopamine-lesioned rats. Brain Res.

[80] Klemm WR (1965). Potentiation of animal “hypnosis” with low levels of electric current. Anim. Behav.

[81] Klemm WR (1983). Experimental catalepsy: influences of cholinergic transmission in restraint-induced catalepsy. Experientia.

[82] Küfferle B, Tauscher J, Asenbaum S, Vesely C, Podreka I, Brücke T, Kasper S (1997). IBZM SPECT imaging of striatal dopamine-2 receptors in psychotic patients treated with the novel antipsychotic substance quetiapine in comparison to clozapine and haloperidol. Psychopharmacology (Berl.).

[83] Küstner U, Müller-Oerlinghausen B (1985). The handwriting test as an aid to neuroleptic dose. Int. J. Clin. Pharmacol. Ther. Toxicol.

[84] Lehmann J, Langer SZ (1983). The striatal cholinergic interneuron: synaptic target of dopaminergic terminals?. Neuroscience.

[85] Leysen JE, Niemegeers CJE, Tollenaere JP, Laduron PM (1978). Serotonergic component of neuroleptic receptors. Nature.

[86] Leysen J-E, Gommeren W, Eens A, de Chaffoy de Courcelles D, Stoof JC, Janssen PAJ (1988). Biochemical profile of risperidone, a new antipsychotic. J. Pharmacol. Exp. Ther.

[87] Lindenmayer JP, Smith D, Katz I (1984). Radioreceptor assay of neuroleptics in refractory chronic schizophrenic patients. J. Clin. Psychiatry.

[88] Lloyd KG, Mohler H, Heitz P, Bartholini G (1975). Distribution of choline acetyltransferase and glutamate decarboxylase within the substantia nigra and in other brain regions from control and parkinsonian patients. J. Neurochem.

[89] Loopuijt LD (1989). Distribution of dopamine D-2 receptors in the rat striatal complex and its comparison with acetylcholinesterase. Brain Res. Bull.

[90] Marder SR, Van Putten T, Mintz J, McKenzie J, Lebell M, Faltico G, May PRA (1984). Costs and benefits of two doses of fluphenazine. Arch. Gen. Psychiatry.

[91] McEvoy JP, Stiller RL, Farr R (1986). Plasma haloperidol levels drawn at neuroleptic threshold doses: a pilot study. J. Clin. Psychopharmacol.

[92] McEvoy JP, Butner NC, Hogarty GE, Steingard S (1991). Optimal dose of neuroleptic in acute schizophrenia: a controlled study of neuroleptic threshold and higher haloperidol dose. Arch. Gen. Psychiatry.

[93] McGraw CP, Klemm WR (1973). Genetic differences in susceptibility of rats to the immobility reflex: “Animal hypnosis”. Behav. Genet.

[94] McQuade RD, Duffy RA, Coffin VL, Barnett A (1992). *In vivo* binding to dopamine receptors: a correlate of potential antipsychotic activity. Eur. J. Pharmacol.

[95] Melkersson K (2005). Differences in prolactin elevation and related symptoms of atypical antipsychotics in schizophrenic patients. J. Clin. Psychiatry.

[96] Meltzer HY, Matsubara S, Lee J-C (1989). Classification of typical and atypical antipsychotic drugs on the basis of dopamine D-1, D2 and serotonin-2 pKi values. J. Pharmacol. Exp. Ther.

[97] Miller R (1976). Schizophrenic psychology, associative learning and the role of forebrain dopamine. Med. Hypothese.

[98] Miller R (1984). Major psychosis and dopamine: controversial features and some suggestions. Psychol. Med.

[99] Miller R (1987). The time course of neuroleptic action for psychosis: role of learning processes and implications for concepts of psychotic illness. (A Review). Psychopharmacology (Berl.).

[100] Miller R (1993). Striatal dopamine in reward and attention: a system for understanding symptomatology of acute schizophrenia and mania. Int. Rev. Neurobiol.

[101] Miller R (1997). Dose-response relationships for the antipsychotic effects and Parkinsonian side-effects of typical neuroleptic drugs: Practical and theoretical implications. Prog. Neuropsychopharmacol. Biol. Psychiatry.

[102] Miller R (2008a). A Theory of the Basal Ganglia and their Disorders. (Conceptual Advances in Brain Research series).

[103] Miller R (2008b). A Neurodynamic Theory of Schizophrenia and Related Disorders. Morrinsville, North Carolina, Lulu Enterprises, Inc(details: www.robertmiller-octspan.co.nz).

[104] Miller R, Chouinard G (1993). Loss of striatal cholinergic neurons as a basis for tardive and L-Dopa-induced dyskinesias, neuroleptic-induced supersensitivity psychosis and refractory schizophrenia. Biol. Psychiatry.

[105] Miller R, Wickens JR, Beninger RJ (1990). Dopamine D-1 and D-2 receptors in relation to reward and performance: a case for the D-1 receptor as a primary site of therapeutic action of neuroleptic drugs. Prog. Neurobiol.

[106] Mizrahi R, Kiang M, Mamo DC, Arenovich T, Bagby RM, Zipursky RB, Kapur S (2006). The selective effect of antipsychotics on the different dimensions of the experience of psychosis in schizophrenia spectrum disorders. Schizophr. Res.

[107] Mones RJ (1972). L-Dopa-induced dyskinesias in normal rhesus monkey. Mt. Sinai J. Med.

[108] Mones RJ, Barbeau A, Chase TN, Paulson GE (1973). Experimental Dyskinesias in Normal Rhesus Monkey.

[109] Mones RJ, Elizan TS, Siegel G (1971). Analysis of L-DOPA induced dyskinesias in 51 patients with parkinsonism. J. Neurol. Neurosurg. Psychiatry.

[110] Nisenbaum ES, Stricker EM, Zigmon MJ, Berger TW (1986). Long-term effects of dopamine-depleting lesions on spontaneous activity of type II striatal neurons: relation to behavioural recovery. Brain Res.

[111] Nishikawa T, Tsuda A, Tanaka M, Koga I, Ushida Y (1982). Prophylactic effects of neuroleptics in symptom-free schizophrenics: roles of dopaminergic and noradrenergic blockers. Biol. Psychiatry.

[112] Nordberg A, Nyberg P, Windblad B (1985). Topographic distribution of choline acetyltransferase activity and muscarinic and nicotinic receptors in Parkinson brains. Neurochem. Pathol.

[113] Nordström AL, Farde L, Wiesel FA, Forslund K, Pauli S, Halldin C, Uppfeldt G (1992). Central D2-dopamine receptor occupancy in relation to antipsychotic drugs effects a double-blind PET study of schizophrenic patients. Biol. Psychiatry.

[114] Nyberg S, Eriksson B, Oxenstierna G, Halldin C, Farde L (1999). Suggested minimal effective dose of risperidone based on PET-measured D2 and 5-HT2a receptor occupancy in schizophrenic patients. Am. J. Psychiatry.

[115] Olianas MC, Adem A, Karlsson E, Onali P (1996). Rat striatal muscarinic receptors coupled to the inhibition of adenylyl cyclase activity: potent block by the selective M4 ligand muscarinic toxin 3 (MT3). Br. J. Pharmacol.

[116] Olianas MC, Adem A, Karlsson E, Onali P (1998). Identification of rat brain muscarinic M4 receptors coupled to cyclic AMP using the selective antagonist muscarinic toxin 3. Eur. J. Pharmacol.

[117] Olsson H, Farde L (2005). Half-life of receptor occupancy - a meaningless concept. Int. J. Neuropsychopharmacol.

[118] Onali P, Olianas MC (2002). Muscarinic M4 receptor inhibition of dopamine D1-like receptor signalling in rat nucleus accumbens. Eur. J. Pharmacol.

[119] Passero S, Carli G, Battistini N (1981). Depression of cerebral glucose utilization during animal hypnosis in the rabbit. Neurosci. Lett.

[120] Pfeiffer CC, Jenney EH (1957). The inhibition of conditioned responses and the counteraction of schizophrenia by muscarinic stimulation of the brain. Ann. N.Y. Acad. Sci.

[121] Pierelli F, Adipietro A, Soldati G, Fattaposta F, Pozzessere G, Scopetti C (1998). Low dosage clozapine effects on L-DOPA-induced dyskinesia in parkinsonian patients. Acta Neurol. Scand.

[122] Pilowsky LS, Costa DC, Ell PJ, Murray RM (1993). Anti-psychotic medication, D2 dopamine receptor blockade and clinical response: a 123I IBZM SPET (single photon emission tomography) study. Psychol. Med.

[123] Qian. Y, Hitzemann B, Hitzemann R (1992). D1 and D2 dopamine receptor distribution in the neuroleptic nonresponsive and neuroleptic responsive lines of mice; a quantitative receptor autoradiographic study. J. Pharmacol. Exp. Ther.

[124] Rabey JM, Treves TA, Neufeld MY, Orlov E, Korczyn AD (1995). Low-dose clozapine in the treatment of levodopa-induced mental disturbances in Parkinson's disease. Neurology.

[125] Rasmussen T, Fink-Jensen A, Sauerberg P, Swedberg MD, Thomsen C, Sheardown MJ, Jeppsen L, Calligaro DO, DeLapp NW, Whitesitt C, Ward JS, Shannon HE, Bymaster FP (2001). The muscarinic receptor agonist BuTAC, a novel potential antipsychotic, does not impair learning and memory in mouse passive avoidance. Schizophr. Res.

[126] Reisine TD, Fields JZ, Yamamura HI (1977). Neurotransmitter receptor alterations in Parkinson's disease. Life Sci.

[127] Richelson E, Souder T (2000). Binding of antipsychotic drugs to human brain receptors. Focus on newer generation compounds. Life Sci.

[128] Roth BL, Tandra S, Burgess LH, Sibley DR, Meltzer HY (1995). D4 dopamine receptor binding affinity does not distinguish between typical and atypical antipsychotic drugs. Psychopharmacology (Berl.).

[129] Rowell FF, Hui SM, Fairburn AF, Eccleston D (1981). Total and free serum haloperidol levels in schizophrenic patients and the effect of age, thioridazine and fatty acids on haloperidol serum protein binding *in vitro*. Br. J. Clin. Pharmacol.

[130] Ruggieri S, De Pandis MF, Bonamartini A, Vacca L, Stocchi F (1997). Low dose clozapine in the treatment of dopaminergic psychosis in Parkinson's disease. Clin. Neuropharmacol.

[131] Rylander G (1972). Psychoses and the punding and choreiform syndromes in addiction to central stimulant drugs. Psychiatr. Neurol. Neurochir.

[132] Sanchez-Lemus E, Arias-Montano JA (2006). M1 muscarinic receptors contribute to, whereas M4 receptors inhibit, dopamine D1 receptor-induced [3H]-cyclic AMP accumulation in rat striatal slices. Neurochem. Res.

[133] Sassin JF, Meldrum BS, Marsden CD (1975). Drug-Induced Dyskinesias in Monkeys. Primate Models of Neurological Disorders. (Adv. Neurol., Vol 10).

[134] Sassin JF, Taub S, Weitzman ED (1972). Hyperkinesia and changes in behaviour produced in normal monkeys by L-dopa. Neurology.

[135] Scatton B (1982). Further evidence for the involvement of D2 but not D1 dopamine receptors in dopaminergic control of striatal cholinergic transmission. Life Sci.

[136] Schweitzer BI, Bacopoulos NG (1983). Reversible decrease in dopaminergic 3H-agonist binding after 6-hydroxydopamine and irreversible decrease after kainic acid. Life Sci.

[137] Seeman P (1980). Brain dopamine receptors. Pharmacol. Rev.

[138] Seeman (2002). Atypical antipsychotics: mechanism of action. Can. J. Psychiatry.

[139] Sesack SR, Aoki C, Pickel VM (1994). Ultrastructural localization of D2 receptor-like immunoreactivity in midbrain dopamine neurons and their striatal target. J. Neurosci.

[140] Shannon HE, Rasmussen K, Bymaster FP, Hart JC, Peters SC, Swedberg MDB, Jeppesen L, Sheardown MJ, Sauerberg P, Fink-Jensen A (2000). Xanomeline, an M1/M4 preferring muscarinic cholinergic receptor agonist, produced antipsychotic-like activity in rats and mice. Schizophr. Res.

[141] Shen W, Hamilton SE, Nathanson NM, Surmeier JD (2005). Cholinergic suppression of KCNQ channel currents enhances excitability of striatal medium spiny neurons. J. Neurosci.

[142] Simpson GM, Krako L, Mattke D, St. Phard GS (1970). A controlled comparison of the treatment of schizophrenic patients when treated according to the neuroleptic threshold or by clinical judgement. Acta Psychiatr. Scand. Suppl.

[143] Stille G, Lauener H, Eichenberger E (1971). The pharmacology of 8-chloro-11-(4-methyl-1-piperaninyl) 5-H-dibenzo[b,e]di-azepine(clozapine). FARMACO.

[144] Stoof JC, De Boer T, Sminia P, Mulder AH (1982). Stimulation of D2-dopamine receptors in rat neostriatum inhibits the release of acetylcholine and dopamine but does not affect the release of gamma-aminobutyric acid, glutamate or serotonin. Eur. J. Pharmacol.

[145] Stoof JC, Verheijden PMFH, Leysen JE (1987). Stimulation of D-2 receptors in rat nucleus accumbens slices inhibits dopamine and actylcholine release, but not cyclic AMP formation. Brain Res.

[146] Sullivan RJ, Andres S, Caleb O, Miles W, Kydd R (2007). The effects of an indigenous muscarinic drug, betal nut (Areca catechu), on the symptoms of schizophrenia: a longitudinal study in Palau, Micronesia. Am. J. Psychiatry.

[147] Svendsen CN, Froimowitz M, Hrbek C, Campbell A, Kula N, Baldessarini RJ, Cohen BM, Babb S, Teicher MH, Bird ED (1988). Receptor affinity, neurochemistry and behavioral characteristics of the enantiomers of thioridazine: evidence for different stereoselectivities at d1 and d2 receptors in rat brain. Neuropharmacology.

[148] Tauscher J, Küfferle B, Asenbaum S, Tauscher-Wisniewski S, Kasper S (2002). Striatal dopamine-2 receptor occupancy as measured with [I123]iodobenzamide and SPECT predicted the occurrence of EPS in patients treated with atypical antipsychotic and haloperidol. Psychopharmacology (Berl.).

[149] Tauscher J, Hussain T, Agid O, Verhoeff NPLG, Wilson AA, Houle S, Remington G, Zipursky RB, Kapur S (2004). Equivalent occupancy of dopamine D1 and D2 receptors with clozapine: differentiation from other atypical antipsychotics. Am. J. Psychiatry.

[150] The French Clozapine Parkinson Study Group (1999). Clozapine in drug-induced psychosis in Parkinson's disease. Lancet.

[151] The Parkinson Study Group (1999). Low-dose clozapine for the treatment of drug-induced psychosis in Parkinson's disease. N. Engl. J. Med.

[152] Togasaki DM, Tan L, Protell P, Di Monte DA, Quik M, Langston JW (2001). Levodopa induces dyskinesias in normal squirrel monkeys. Ann. Neurol.

[153] Wang Z, Kai L, Ronesi J, Yin HH, Ding J, T katch T, Lovinger DM, Surmeier DJ (2006). Dopaminergic control of corticostriatal long-term synaptic depression in medium spiny neurons is mediated by cholinergic interneurons. Neuron.

[154] Wiesel F-A, Farde L, Nordström A-L, Sedvall G (1990). Central D1- and D2-receptor occupancy during antipsychotic drug treatment. Prog. Neuropsychopharmacol. Biol. Psychiatry.

[155] Wolkin A, Barouche F, Wolf AP, Rotrosen J, Fowler JS, Shiuo C-Y, Cooper TB, Brodie JD (1989). Dopamine blockade and clinical response: evidence for two biological subgroups of schizophrenia. Am. J. Psychiatry.

[156] Wong DT, Bymaster FP, Reid LR, Fuller RW, Perry KW, Kornfeld EC (1983). Effect of a stereospecific D2-dopamine agonist on acetylcholine concentration in corpus striatum in rat brain. J. Neural Transm.

[157] Wunderink L, Nienhuis FJ, Sytema S, Slooff CJ, Knegtering R, Wiersma D (2007). Guided discontinuation versus maintenance treatment in remitted first-episode psychosis: relapse rates and functional outcome. J. Clin. Psychiatry.

[158] Yan Z, Flores-Hernandez J, Surmeier JD (2001). Coordinated expression of muscarinic receptor mRNAs in striatal medium spiny neurons. Neuroscience.

[159] Zeng XP, Le F, Richelson E (1997). Muscarinic M4 receptor activation by some atypical antipsychotic drugs. Eur. J. Pharmacol.

[160] Zhou L-W, Zhang S-P, Connell TA, Weiss B (1993). Cholinergic lesions of mouse striatum induced by AF64A alter D2 dopaminergic behavior and reduced D2 dopamine receptors and D2 dopamine receptor mRNA. Neurochem. Int.

[161] Zimbroff DL, Kane JM, Tamminga CA, Daniel DG, Mack RJ, Wozniak PJ, Sebree TB, Wallin BA, Kashkin KB, Sertindole Study Group (1997). Controlled, dose-response study of sertindole and haloperidol in the treatment of schizophrenia. Am. J. Psychiatry.

